# Prenatal methadone exposure produces functional and molecular alterations in the basolateral amygdala and decreased voluntary ethanol intake in female, but not male offspring

**DOI:** 10.3389/fnbeh.2025.1570951

**Published:** 2025-04-15

**Authors:** Meredith E. Gamble, Michelle Montero, Dana N. Silberstein, Terrence Deak, Elena I. Varlinskaya, Marvin R. Diaz

**Affiliations:** ^1^Department of Psychology, Center for Development and Behavioral Neuroscience, Binghamton University, Binghamton, NY, United States; ^2^Developmental Exposure Alcohol Research Center, Binghamton, NY, United States

**Keywords:** prenatal, methadone, opioid, basolateral amygdala, ethanol, stress

## Abstract

**Introduction:**

A result of the ongoing opioid epidemic has been a significant rise in the rates of opioid use during pregnancy. This includes use of maintenance medications for opioid use disorder (MOUDs), such as methadone, which are the standard of care for pregnant people with an opioid use disorder (OUD). Although the use of MOUDs leads to better neonatal outcomes in exposed offspring compared to those born from individuals with untreated OUD, the pharmacology of MOUDs is similar to misused opioids. Despite the high prevalence of prenatal exposure to opioids, including MOUDs, our understanding of the long-term consequences of these exposures in offspring is limited. Prenatal drug exposure is known to be a risk factor for future substance use disorder and mood disorders, yet, how prenatal opioid exposure influences ethanol intake in adult offspring and associated affective behaviors has not been examined.

**Methods:**

Using a rat model of prenatal methadone exposure (PME), which included twice daily methadone injections from gestational day 3-20, this study assessed ethanol intake in adult offspring and how exposure to forced swim stress (FSS) altered ethanol intake, in addition to examination of depressive-like behavior during the FSS. Given the role of the basolateral amygdala (BLA) in emotion and reward processing, we also conducted patch clamp electrophysiology experiments from BLA neurons to investigate changes in synaptic transmission and gene expression of neuromodulatory systems that are known to influence emotion and reward processing.

**Results:**

Females with a history of PME consumed less ethanol than control females, with no effects of PME on ethanol intake evident in males. While PME increased immobility during FSS in both males and females, FSS had no effects on ethanol intake. PME increased glutamate transmission and altered dopamine D1, D2, and D3 receptor and mu opioid receptor mRNA in the BLA of females, but not in males.

**Discussion:**

Collectively, this study identified impairments in emotion and reward processing, in addition to alterations in synaptic function and gene expression in the BLA of females with a history of PME, supporting previous findings from our lab demonstrating that female offspring are more sensitive to the long-term effects of PME.

## Introduction

Opioid misuse and the incidence of opioid use disorder (OUD) has significantly risen over the past couple of decades (Spencer et al., 2024). Maintenance medications for OUD (MOUDs), such as methadone and buprenorphine, can facilitate abstinence and recovery from OUD, making this therapeutic an important form of harm reduction (Taylor et al., 2021). However, MOUDs are also approved and recommended for pregnant people with an OUD (Caritis and Panigrahy, 2019; Haight et al., 2018; Rodriguez and Klie, 2019), since there is an extensive literature supporting the use of MOUDs during pregnancy as a means to improve neonatal outcomes by reducing symptoms of Neonatal Opioid Withdrawal Syndrome (NOWS). Despite the beneficial effects of MOUDs, long-term consequences of MOUD use during pregnancy are concerning given that MOUDs are agonists at the same receptors that bind misused opioids. Evidence of this concern is becoming increasingly clear as there has been a rise in clinical and preclinical studies demonstrating potentially long-term effects of MOUDs in prenatally-exposed offspring (Aslaksen et al., 2024; Byrnes and Vassoler, 2018; Chen et al., 2015; Daly et al., 2012; Farid et al., 2008; Gamble and Diaz, 2023; Gamble et al., 2022; Hasan et al., 2024; Levine et al., 2021; Monnelly et al., 2019; Sahid et al., 2025; Spowart et al., 2023). While harm reduction is critical for pregnant people with OUD, there remains a large gap in our knowledge regarding the long-term effects of MOUD exposure in offspring.

Among long-term effects of prenatal opioid exposure, including exposure to MOUDs, increased addictive-related behaviors and negative affective behaviors have been reported repeatedly. In humans, a history of prenatal exposure to opioids is associated with increased emotional processing deficits, including anxiety and depression, as well as alterations in social behaviors (Baldacchino et al., 2014; Baldacchino et al., 2012; de Cubas and Field, 1993; Jaekel et al., 2021; Levine et al., 2021). Animal research demonstrates alterations in emotion processing evident in humans, for example, prenatal exposure to opioids, including MOUDs, increased anxiety-like behavior and reduced social interaction in rat offspring (Byrnes and Vassoler, 2018; Chen et al., 2015; Daly et al., 2012; Farid et al., 2008; Gamble and Diaz, 2023). Interestingly, prenatal opioid exposure of laboratory rodents has been shown to increase addiction-like behaviors to a variety of substances (see reviews Abu and Roy, 2021; Grecco and Atwood, 2020), including alcohol (Grecco et al., 2022a). Importantly, there is a well-established interplay between negative affect and addiction susceptibility, whereby negative affect can drive addiction-related behaviors, and vice-versa (Koob, 2015, 2022; Koob and Schulkin, 2019; Koob and Volkow, 2016). However, it is still unclear whether this relationship exists following prenatal opioid exposure. Importantly, knowledge of prenatal MOUD exposure effects may help guide targeted interventions to prevent the emergence of later substance misuse among affected offspring.

The basolateral amygdala (BLA) is involved in emotion and reward processing (Janak and Tye, 2015). Specifically, the BLA receives cortical and sensory inputs, which are locally processed and transmitted to downstream structures within the anxiety circuitry to drive both positive and negative emotions. Increased glutamatergic transmission within the BLA is hypothesized to drive BLA pyramidal neuron activity, which is correlated with negative affective behaviors, including anxiety and depression, as well as alterations in reward processing (Janak and Tye, 2015; Qin et al., 2021; Wassum and Izquierdo, 2015; Zhang et al., 2019; Zhang and Rosenkranz, 2012). Interestingly, there is recent evidence that human infants with prenatal opioid exposure showed increased functional connectivity between the amygdala and prefrontal cortex (Radhakrishnan et al., 2020) and smaller amygdalar volumes (Aslaksen et al., 2025), suggesting that the amygdala may be a target of prenatal opioid exposure contributing to impairments in emotion and reward processing. A recent preclinical study also identified alterations in amygdala activity in late-adolescent offspring with a history of prenatal methadone exposure (PME) (Grecco et al., 2022b). Despite these observations, long-term effects of prenatal opioid exposure on BLA function are not yet known.

Using a rat model of PME in which pregnant dams were exposed to 5–7 mg/kg twice daily on gestational days (G) 3–20, we recently reported that adult female PME offspring demonstrated heightened fear conditioning relative to control females, whereas male offspring were unaffected by PME (Gamble and Diaz, 2023). These female-specific effects were consistent with various other neurogenic, neurophysiological, and neurobehavioral alterations that we have previously reported (Gamble and Diaz, 2023; Gamble et al., 2022). Given our understanding of prenatal opioid exposure’s effects, particularly PME, the objective of the current study was to examine how PME affects (1) ethanol intake, (2) how later stress affects ethanol intake, (3) acute stress reactivity, and (4) BLA glutamatergic transmission in adult offspring. Additionally, we explored potential neuromodulatory systems within the BLA that may be disrupted by PME to identify potential mechanisms that may drive PME’s effects on BLA dysfunction and associated behavioral alterations.

## Methods

### Animals

Male and female Sprague–Dawley rats, originating from Envigo (Indianapolis, IN) were bred in-house. At weaning on postnatal day (P) 21, same-sex littermates were housed 2–3 per cage and randomly assigned to an experiment. Rats received 5L0D PicoLab Laboratory Rodent Diet and water ad libitum and were maintained on a 12 h light/dark cycle (lights on at 07:00). All procedures and protocols were in accordance with the National Institutes of Health guidelines for animal care using protocols approved by Binghamton University Institutional Animal Care and Use Committee.

### Breeding and prenatal methadone exposure

Breeding and exposure procedures were identical to those described previously by our group (Gamble and Diaz, 2023; Gamble et al., 2022). Briefly, 2 drug naïve, nulliparous females and 1 male were housed together for up to 4 days. Daily vaginal swabs were conducted to detect the presence of sperm. Pregnancy was confirmed on the first day of detectable sperm, designated gestational day (G) 1, at which point the pregnant female was single-housed and randomly assigned to an exposure condition (control or methadone). Pregnant and lactating dams received Purina Lab Diet 5008C33 and water ad libitum. PME began on G3 with twice daily subcutaneous injections, 12 h apart (07:00 and 19:00), of 5 mg/kg of either methadone dissolved in sterile water (5 mg/mL solution) or sterile water alone (control group). From G4-20, the dose was increased to 7 mg/kg. Due to common side effects of methadone exposure in rats, particularly gnawing and pica, all dams (methadone and water-injected) were placed in a cage with no bedding (but with access to food and water) for 3 h following each injection to prevent ingestion of bedding and nesting material. Dams were returned to their home cages after 3 h until the next injection. 1% silver sulfadiazine topical cream was used to treat minor skin irritation due to injections where necessary. Dam weights were taken daily to determine administration volume; data on dam weight has been previously published (Gamble et al., 2022).

After the G20 exposure, dams were not disturbed and were allowed to give birth. After giving birth, pups were counted, weighed, and culled to 12 (equal male:female ratio) on P2. Pups were also weighed on P7 and P12, and were weaned on P21. Following weaning, offspring were allowed to age to adulthood (P70+) and randomized to an experiment; each experiment (voluntary ethanol intake/forced swim stress, electrophysiology, and PCR) was conducted in separate subsets of animals. Each experiment had no more than 2 pups per sex from the same litter.

### Voluntary ethanol intake

A limited-access voluntary ethanol intake test was used to assess the effect of PME on ethanol consumption (control females = 7 litters; PME females = 5 litters; control males = 5 litters; PME males = 8 litters). As previously reported (Gamble and Diaz, 2020), ethanol intake sessions occurred every Monday, Wednesday, and Friday evening (19:00) for 3 weeks (total of 9 sessions). Briefly, animals were transported to a testing room, weighed, and placed in individual clean cages. A single drinking bottle containing sweetened ethanol solution (10% ethanol +3% sucrose +0.125% saccharin) was place on each cage for 30 min. The sweetened solution was used to increase palatability, consistent with previous methodologies for voluntary ethanol intake in rats (Broadwater et al., 2013; Walker et al., 2008). Drinking bottles were weighed before and after testing to calculate g/kg of ethanol consumed for each animal per session using the animals’ weight measured before each session. Daily intake was averaged per week and cumulative total intake over the 3 weeks was also calculated.

**Figure 1:**
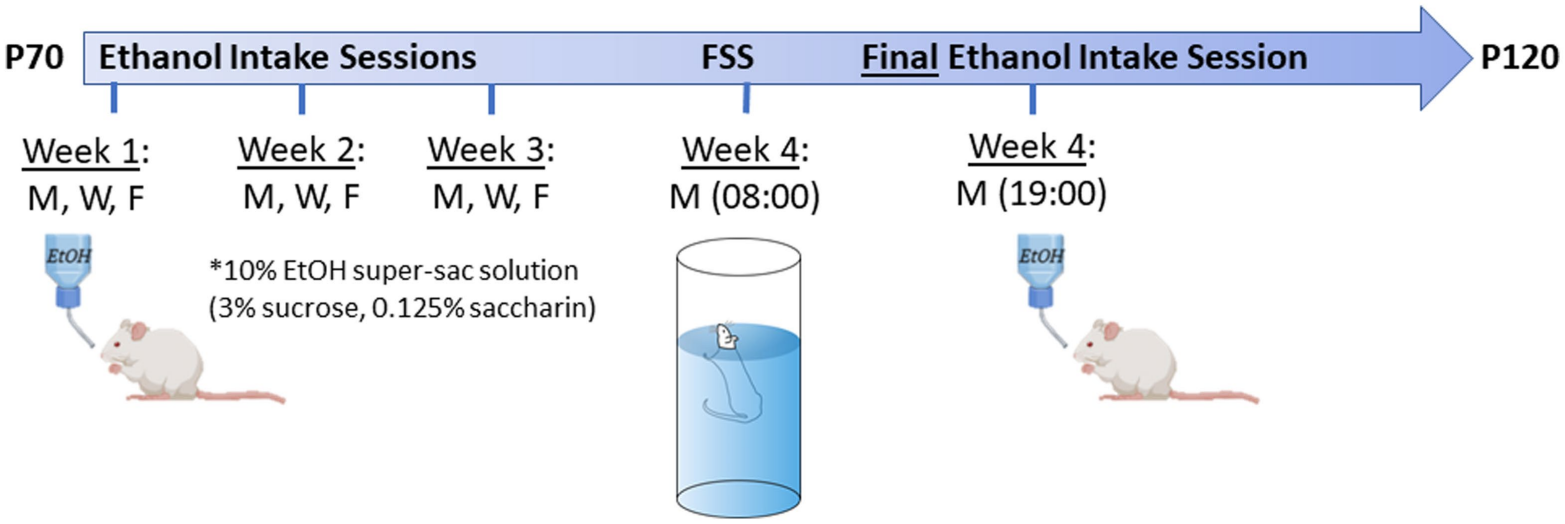
Experimental time-line for ethanol intake + forced swim stress (FSS) test in adult offspring. Testing age range was P70-120. Animals went through 3 weeks of 30 min limited-access voluntary ethanol intake sessions (10% ethanol +3% sucrose +0.125% saccharin) on Monday, Wednesday, and Friday at 19:00. On the Monday of the 4th week, all animals went through a FSS test ~09:00, followed by a final ethanol intake session at 19:00. Images were created using BioRender.

The final ethanol intake session was conducted on the 4th Monday as described above, 10–12 h after the forced swim stress (see below). See Figure 1 for a schematic depiction of the ethanol intake and forced swim stress time-course.

### Forced swim stress

The Monday after the 3rd week of voluntary ethanol intake, rats underwent a single 10-min session of forced swim stress (FSS) in the morning at 08:00 as previously reported (Gamble and Diaz, 2020). Briefly, subjects were transported to the testing room and placed in individual polycarbonate cylinders (height = 45.72 cm, diameter = 20.32 cm) filled with water to 25 cm deep at 25°C. The 10 min sessions were video recorded for later scoring analysis. After the 10-min session, rats were dried with a towel and placed in a new cage for 30 min to allow further drying, and were then returned to their home cage and colony.

The 10-min FSS videos were scored by a blinded experimenter. Using the criteria discussed in Deak and Larish (2006), time to become immobile and total immobile time were calculated for each animal. Time to become immobile was defined as the seconds from when the animal entered the water to the time that they first became immobile, with immobility defined as the animal floating and only making minimal movements required to keep their head above water.

### Drugs and chemicals

All chemicals used in electrophysiology and ethanol intake experiments were purchased from Sigma-Aldrich (St. Louis, MO), unless otherwise noted. Gabazine was purchased from Tocris/R&D Systems (Bristol, UK). Ethanol was purchased from Pharmco by Greenfield Global (Toronto, CA).

### Whole cell patch-clamp electrophysiology

As previously described (Przybysz et al., 2017), using a different subset of animals (not behaviorally tested; control females = 7 litters; PME females = 8 litters; control males = 6 litters; PME males = 4 litters), P70-120 rats were anesthetized with 3% isoflurane and quickly decapitated. Brains were extracted and immersed in ice cold oxygenated (95% O_2_, 5% CO_2_) sucrose artificial cerebrospinal fluid (ACSF) cutting solution containing (in mM): sucrose (22), KCl (2), NaH_2_PO_4_ (1.3), NaHCO_3_ (26), glucose (10), MgSO_4_ (12), CaCl_2_ (0.2), and ketamine (0.43). 300 μm brain slices containing the BLA were made using a Vibratome (Leica Microsystems, Bannockburn, IL). Slices were incubated in oxygenated ACSF containing (in mM): NaCl (125), KCl (2), NaH_2_PO_4_ (1.3), NaHCO_3_ (26), glucose (10), MgSO_4_ (1), CaCl_2_ (2), and ascorbic acid (0.4) and allowed to recover for at least 40 min at 34°C before recording. Slices remained at 34°C and all experiments were performed within 4 h of slice preparation.

Following incubation, slices were transferred to a recording chamber superfused with oxygenated ACSF at 32°C at 3 mL/min. Recordings were made from pyramidal neurons within the BLA which were visualized using infrared-differential interference contrast microscopy (Olympus America, Center Valley, PA). Pyramidal neurons were visually identified based on morphology, and also neurophysiologically based on capacitance (>150 pF). Spontaneous excitatory postsynaptic current (sEPSC) recordings were collected with patch pipettes filled with K-gluconate internal solution, containing (in mM): K-gluconate (120), KCl (15), EGTA (0.1), HEPES (10), MgCl_2_ (4), Mg-ATP (4), Na-GTP (0.3), and phosphocreatine (7), with a pH of 7.3 and osmolarity of 295–305 mOsm. sEPSCs were pharmacologically isolated using the GABA_A_ receptor antagonist gabazine (10 μM) while clamping the voltage at −70 mV. Neurons were allowed to equilibrate for at least 5 min before sEPSC recording began. We recorded at least 2 min of sEPSCs. Access resistance was monitored throughout each recording, and neurons with a >20% change in access resistance were excluded from analysis. Data were acquired with a MultiClamp 700B (Molecular Devices, Sunnyvale, CA) at 10 kHz, filtered at 1 kHz, and stored for later analysis using MiniAnalysis (Synaptosoft).

### RNA extraction and real time RT-PCR

In a different subset of animals (not behaviorally test; control females = 4 litters; PME females = 4 litters; control males = 5 litters; PME males = 4 litters), rats were euthanized by rapid decapitation under deep anesthesia (3% isoflurane). Following decapitation, brains were quickly extracted and flash frozen in 2-methylbutane and stored at −80°C. The BLA was micro-punched in a cryostat and stored at −80°C until RNA extractions. Tissue punches were extracted with 500 μL Trizol ^®^ RNA reagent and homogenized using Qiagen Tissue-Lyser II ^™^ (Qiagen, Valencia, CA) for 2–4 min at 20 Hz to ensure sufficient homogenization of samples. Total cellular RNA was extracted using Qiagen RNeasy Mini Kits and separated from the supernatant using a chloroform extraction at 4°C. Equal volume of 70% ethanol was added to the collected RNA and purified through RNeasy mini columns. Columns were washed and eluted with 30 μL of RNase-free water (65°C). RNA yield and purity were determined using a NanoDrop 2000 spectrophotometer (NanoDrop, Wilmington, DE). RNA was stored at −80°C prior to cDNA synthesis. cDNA synthesis was conducted on 0.3–1.0 μg of normalized total RNA from each sample using QuantiTect Reverse Transcription kit (catalog # 205313, Qiagen, Valencia, CA), that included a DNase treatment step to remove any residual genomic DNA contamination. Probed cDNA amplification was performed in a 20 μL reaction and run in triplicate in a 384-well plate (BioRad Laboratories) using a RioRad CFX 384 Real Time System C1000 Thermal Cycler (BioRad Laboratories). Relative gene expression was quantified using the delta (2-ΔCT) method relative to the same-sex control. Because housekeeper genes often show fluctuation across experimental groups, we included GAPDH as a separate target gene to demonstrate stability of overall gene expression rather than formally treating GAPDH as a mathematically-adjusted “housekeeper” gene for greater transparency. Primer sequences are provided in Table 1.

**Table 1:** Primer sequences used in real time RT-PCR.

Target gene	Primer sequence	Accession number
*GAPDH*	Forward: GTGCCAGCCTCGTCTCATAG Reverse: AGAGAAGGCAGCCCTGGTAA	NM_017008
*OPRM1*	Forward: GGGCTTGGCGGGAACGACAG Reverse: TGGTCGCTAAGGCGTCTGCC	NM_013071.2
*OPRK1*	Forward: CGCCTTGACTGAATCCCAAC Reverse: GGTCCACGTCCCTGATGTTT	NM_001318742.1
*DRD1*	Forward: CCACTCTCCTGGGCAATACC Reverse: AAAAGGACCCAAAGGGCCAA	NM_012546.3
*DRD2*	Forward: GCAGTCGAGCTTTCAGAGCC Reverse: TCTGCGGCTCATCGTCTTAAG	NM_012547.1
*DRD3*	Forward: CATCCCATTCGGCAGTTTTCAA Reverse: TGGGTGTCTCAAGGCAGTGTCT	NM_017140.2
*DAT*	Forward: TCCTGAAAGGTGTGGGCTTC Reverse: GAGCAGTTGGGGCTATTCCA	NM_012694.2

### Data analyses

Data were analyzed statistically using Prism (GraphPad, San Diego, CA). All data were tested for normal distribution; if data did not pass a test of normality, then non-parametric tests were used. Cumulative ethanol intake and FSS data was first analyzed as a 2-way ANOVA (exposure X sex). Similar to numerous previous studies (Mineur et al., 2022; Radke et al., 2021; Salazar and Centanni, 2024), we found a significant effect of sex in ethanol intake. Based on this, all data were separated by sex and analyzed using either a repeated 2-way ANOVA (exposure × week) for ethanol intake or *t*-test (exposure), electrophysiology, and mRNA expression. In the case of a significant interaction, Sidak’s *post-hoc* analyses were conducted to identify the locus of significance difference. For all statistical comparisons, *p* ≤ 0.05 was considered significant. All data are presented as mean ± SEM.

## Results

### PME reduced ethanol intake in females, but not males

**Figure 2:**
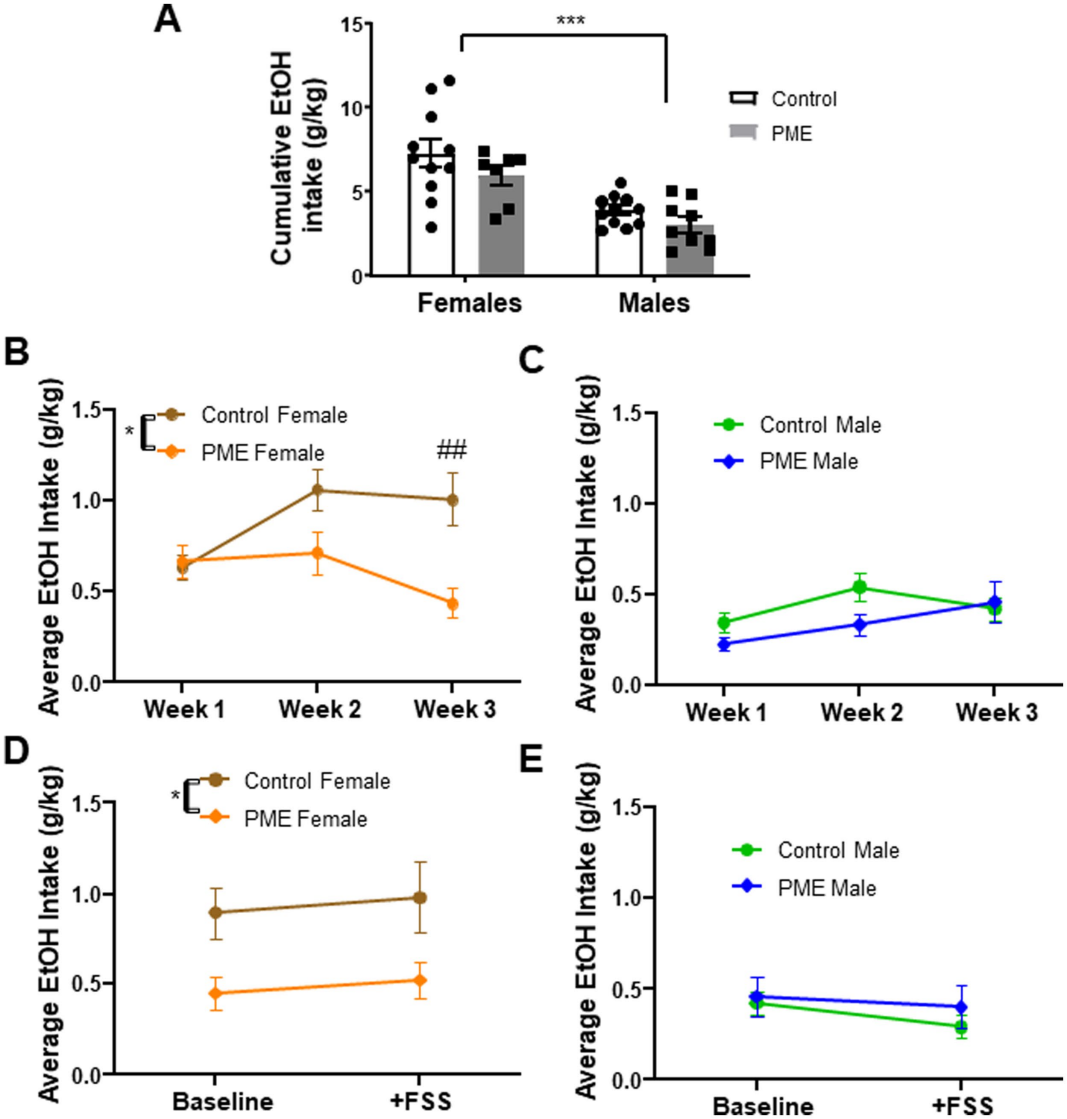
Effects of PME on voluntary ethanol intake in adult offspring. **(A)** Cumulative ethanol intake across 3 weeks—main effect of sex (***p < 0.001) showed that females consumed significantly more ethanol than males. **(B)** Ethanol intake across 3 weeks (averaged by week) in females showed a significant effect of exposure (*p < 0.05) and exposure × week interaction (*p < 0.05); *post-hoc* analysis showed PME females had significantly lower ethanol intake relative to controls on week 3 (^##^*p* < 0.01). **(C)** No significant effects in male ethanol intake across 3 weeks (averaged by week). **(D)** Ethanol intake at baseline (week 3 average) and following FSS in females—no significant effect of stress, with a significant effect of exposure with reduced intake in PME females relative to controls (*p < 0.05). **(E)** No significant effect of stress or exposure in males.

To determine the effect of PME on voluntary ethanol intake, adult offspring went through 3 weeks of limited access ethanol intake sessions. Numerous studies have shown sex differences in ethanol intake in rodents, specifically that females consume more ethanol than males in a variety of ethanol intake paradigms (see recent reviews Mineur et al., 2022; Radke et al., 2021; Salazar and Centanni, 2024). Consistent with the extant literature, we found a significant effect of sex in cumulative ethanol intake over the 3 week period, whereby females consumed significantly more ethanol (Figure 2A: *F* (1, 34) = 28.20, *p* < 0.001), although there was no main effect of exposure (*F* (1, 34) = 3.448, *p* = 0.07) or an interaction (*F* (1, 34) = 0.156, *p* = 0.69). Given the large sex difference, we conducted a more detailed comparison in each sex to determine potential effects of PME as a function of week. Interestingly, in females, a 2-way ANOVA revealed a main effect of exposure (Figure 2B: *F* (1, 16) = 8.198, *p* < 0.05) and an exposure × week interaction (*F* (2, 32) = 5.07, *p* < 0.05), with a strong trend toward a main effect of week (*F* (2, 32) = 3.208, *p* = 0.054). Post-hoc analysis showed that PME females had a significantly lower ethanol intake than controls on week 3 (*p* < 0.01), with no significant differences evident on weeks 1 (*p* = 0.995) or 2 (*p* = 0.073). Conversely, in males there was no effect of exposure (*F* (1, 18) = 2.87, *p* = 0.11) nor an exposure × week interaction (*F* (2, 36) = 1.59, *p* = 0.21), although there was a strong trend toward a main effect of week (*F* (2, 36) = 3.189, *p* = 0.053), suggestive of a subtle increase in ethanol intake over the 3 weeks (Figure 2C).

### PME increased immobility in a forced swim test

**Figure 3:**
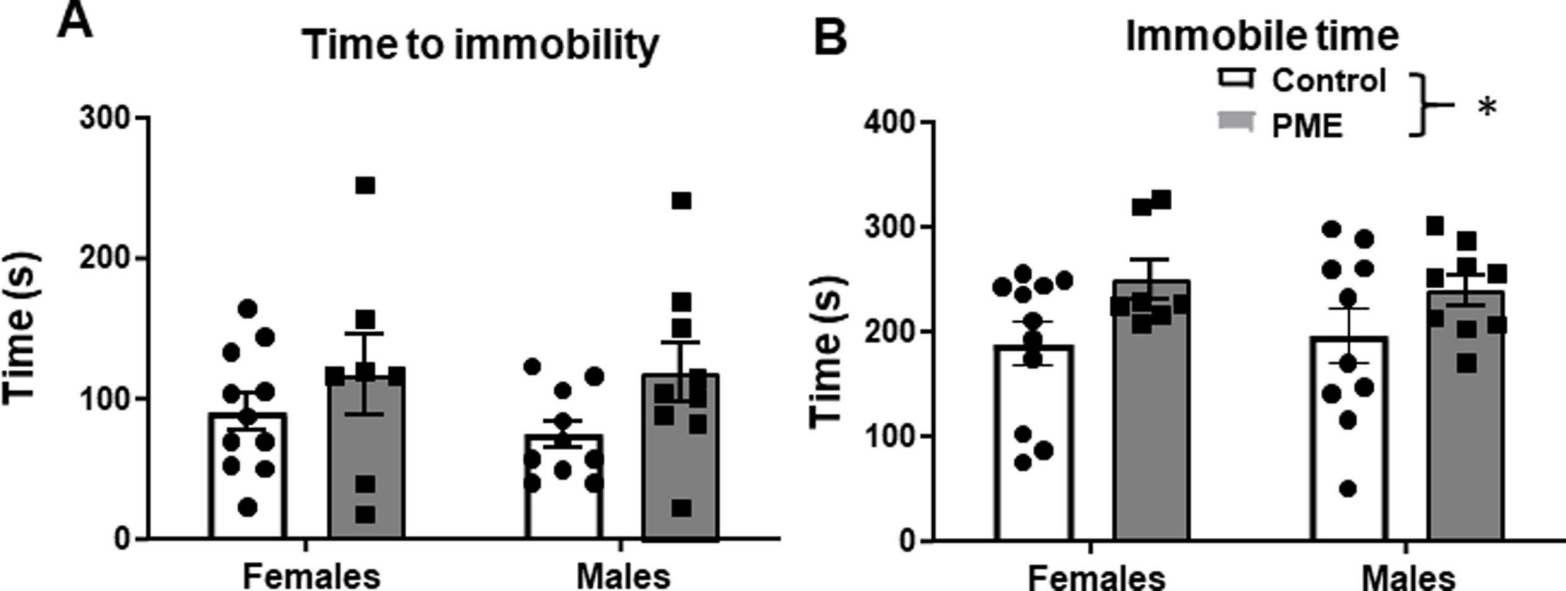
PME increased immobility time in the forced swim stress (FSS) test in adult offspring. **(A)** No effect of sex or exposure on time to first immobile episode. **(B)** PME significantly increased immobility time relative to controls (*p < 0.05), with no effect of sex.

Increased negative affective behaviors, including reactivity to stress, have been reported in offspring with a history of PME. To examine potential effects of PME on acute stress reactivity, after the 3 weeks of voluntary ethanol intake, subjects were put through a single FSS test for 10 min. There was a trend for a significant difference in time to first instance of immobility as a function of exposure (*F* (1, 33) = 3.885, *p* = 0.057), with no effect of sex (*F* (1, 33) = 0.162, *p* = 0.69), or an interaction (*F* (1, 33) = 0.284, *p* = 0.60) (Figure 3A). Time was significantly higher in PME subjects relative to controls (main exposure effect: *F* (1, 33) = 5.718, *p* = 0.023) (Figure 3B). However, there was no sex difference (*F* (1, 33) = 0.003, *p* = 0.96) and no exposure × sex interaction (*F* (1, 33) = 0.189, *p* = 0.66). These findings suggest differences in response to acute stress in the FSS in PME offspring, independent of sex.

### Stress exposure did not affect ethanol intake

Exposure to stress can drive elevations in ethanol intake (Mineur et al., 2022), though these effects have been notoriously variable and depend upon the species tested, the timing and nature of the stress challenge imposed, and the approach for measuring ethanol intake (see Becker et al., 2011 for review). Given that prenatal opioid exposure, particularly PME, can disrupt neural responses to stress exposure, including reducing density of stress-neurons (i.e., corticotropin-releasing factor positive neurons) (Wu et al., 2023), we investigated whether exposure to FSS would differentially alter voluntary ethanol intake in PME offspring. Ten-twelve hours after FSS, subjects were given a single 30-min access ethanol intake session. Intake after FSS was compared to the average intake in week 3 (“baseline”). In females, we found a persistent main effect of exposure (*F* (1, 16) = 5.29, *p* = 0.03), with intake being significantly lower in PME females relative to controls (Figure 2D). However, there was no effect of stress (*F* (1, 16) = 0.408, *p* = 0.53) or an exposure × stress interaction (*F* (1, 16) = 0.004, *p* = 0.94). In contrast, males did not show an effect of exposure (*F* (1, 18) = 0.484, *p* = 0.49), stress (*F* (1, 18) = 2.17, *p* = 0.15), or interaction (*F* (1, 18) = 0.302, *p* = 0.59) (Figure 2E). These data indicate that FSS ~10 h prior to access to ethanol did not affect ethanol intake in either control or PME offspring. Furthermore, PME-induced reductions in ethanol intake in females persist despite exposure to stress.

### PME increases BLA glutamate transmission only in females

**Figure 4:**
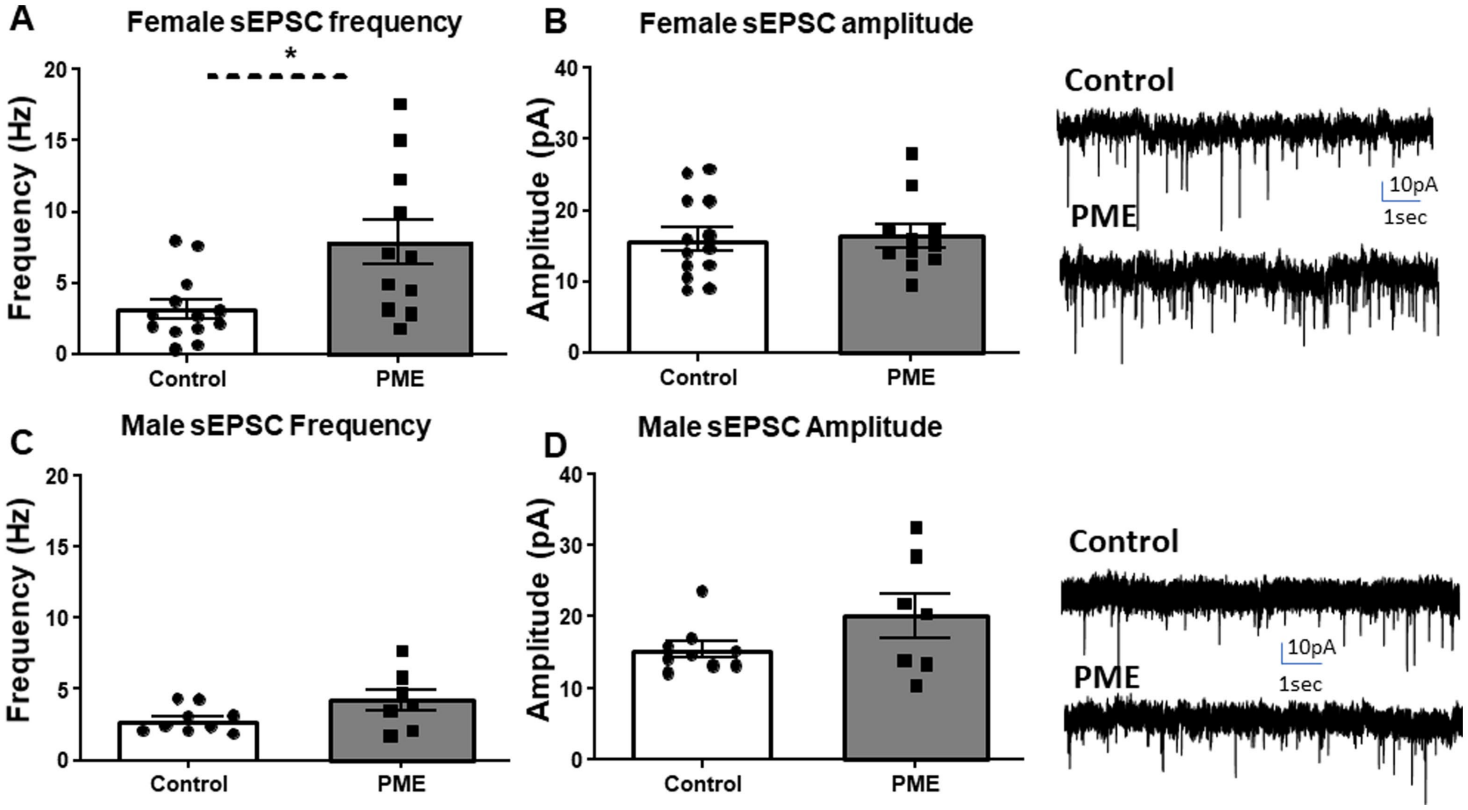
PME altered BLA glutamatergic transmission in females, but not males. PME significantly increased sEPSC **(A)** frequency (*p < 0.05), but not **(B)** amplitude in females—exemplar sEPSC traces from control and PME females (control: *N* = 7 animals, *n* = 13 cells; PME: *N* = 8 animals, *n* = 11 cells). PME did not affect sEPSC **(C)** frequency or **(D)** amplitude in males—exemplar sEPSC traces from control and PME males (control: *N* = 6 animals, 9 cells; PME: *N* = 5 animals, *n* = 7 cells).

The BLA is involved in emotion and reward processing (Janak and Tye, 2015). Given the behavioral changes resulting from PME, we next examined glutamatergic transmission within the BLA to begin to identify potential mechanisms that may drive the observed maladaptive behaviors. Neither membrane capacitance nor membrane resistance differed between control and PME males or females (Table 2). Interestingly, examination of glutamatergic transmission showed a significant increase in sEPSC frequency in PME females relative to controls (Figure 4A: *t* = 2.885, df = 22, *p* = 0.0086), but no difference in sEPSC amplitude (Figure 4B: *t* = 0.16, df = 22, *p* = 0.87). Conversely, neither sEPSC frequency (Figure 4C: *t* = 1.719, df = 14, *p* = 0.12) or amplitude (Figure 4D: Mann–Whitney U = 23, *p* = 0.39) differed between control and PME males. These findings suggest that PME increases glutamatergic transmission in adult females, without affecting adult males.

**Table 2:** Membrane capacitance and resistance (mean ± SEM).

	Female control	Female PME	Male control	Male PME
Capacitance (pF)	280.6 ± 10.33	304.2 ± 11.03	299.5 ± 19.42	311.2 ± 24.17
Resistance (MOhm)	135.9 ± 14.96	113.9 ± 10.44	157.8 ± 22.63	108.4 ± 12.10

### PME altered BLA opioid and dopamine receptor gene expression in females only

**Figure 5:**
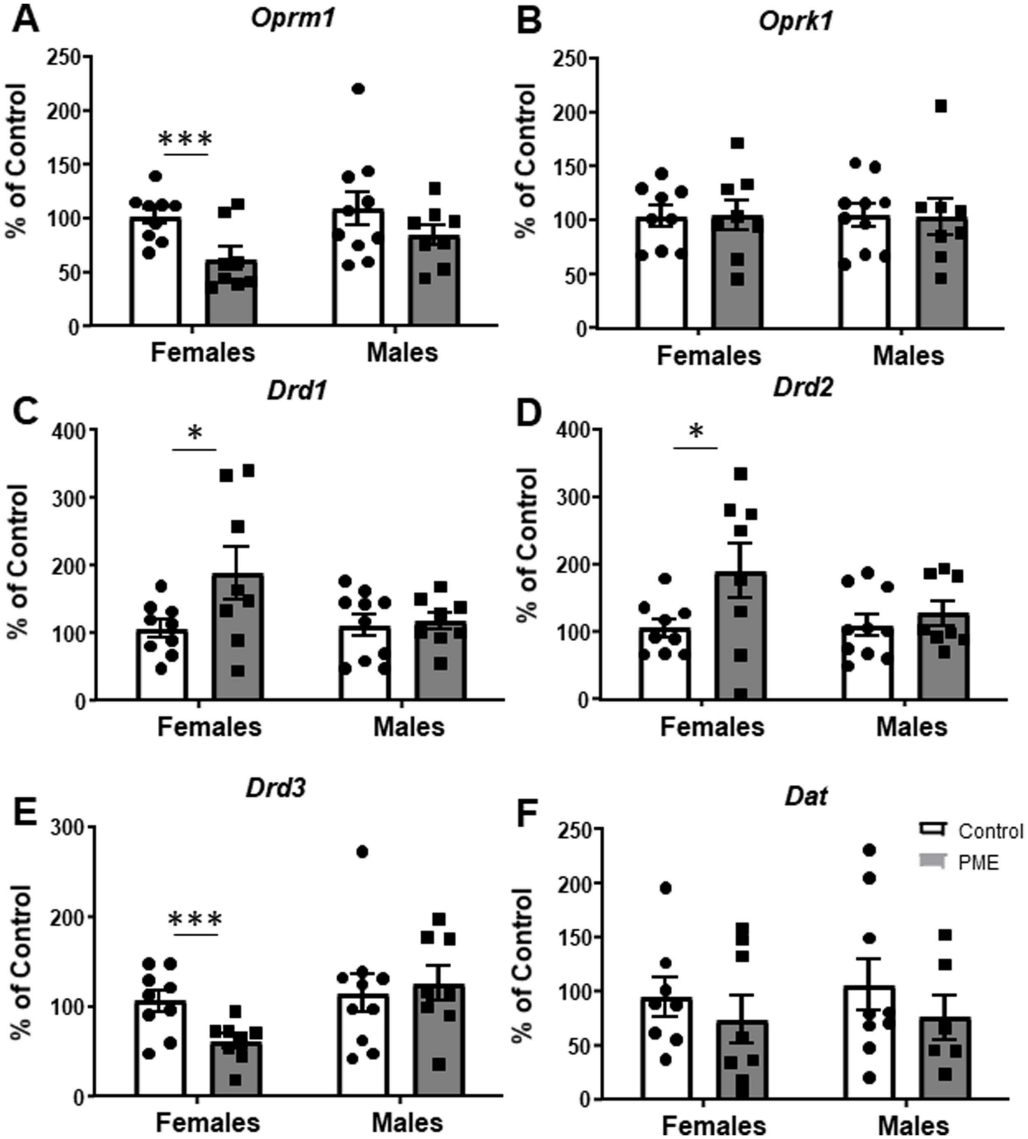
PME disrupted BLA gene expression in females, but not males. PME significantly decreased **(A)** *Oprm1* (***p < 0.001) and **(E)** *Drd3* (***p < 0.001) mRNA in females relative to controls. PME also significantly increased **(C)** *Drd1* (*p < 0.05) and **(D)** *Drd2* (*p < 0.05) mRNA in females relative to controls. PME did not affect **(B)** *Oprk1* or **(F)** *Dat* mRNA in females or any of the genes in males.

We and others have shown that BLA function is modulated by endogenous opioid and dopamine systems through a variety of mechanisms (Diaz et al., 2011; Diaz et al., 2014; Przybysz et al., 2017; Yang et al., 2014). Additionally, chronic morphine exposure in juvenile male rats unmasks a D1R-mediated increase in glutamate transmission in BLA pyramidal neurons, an effect not apparent in controls (Li et al., 2011), whereas withdrawal from chronic heroin exposure downregulates D3R expression within the BLA of adult males (Rosen et al., 2017). Regarding the endogenous opioid system, exposure to prenatal morphine reduces mu-opioid receptor binding in the BLA (Vathy et al., 2003). To begin to identify potential mechanisms that may regulate BLA synaptic function and neurobehavioral alterations resulting from PME, we assessed expression of BLA mu opioid receptor (*Oprm1*), kappa opioid receptor (*Oprk1*), dopamine D1, D2, and D3 receptors (*Drd1*, *Drd2*, *Drd3*, respectively) and dopamine transporter (*Dat*) mRNA. There was no significant effect of PME on GAPDH in either sex, providing evidence for stable cellular gene expression patterns across experimental groups (Table 3 for statistics). Interestingly, we found a significant reduction in *Oprm1* (Figure 5A: *t* = 3.100, df = 15, *p* = 0.007) and *Drd3* (Figure 5E: *t* = 3.030, df = 15, *p* = 0.008) expression in PME females relative to control females (see Table 3 for statistics). We also found a significant increase in *Drd1* (Figure 5C: *t* = 2.109, df = 15, *p* = 0.05) and *Drd2* (Figure 5D: *t* = 2.093, df = 15, *p* = 0.05) in PME females compared to control females. There were no other significant differences in any of the other target genes in females (*Oprk1* - Figure 5B, *Dat* - Figure 5F) nor in any of the target genes in males (see statistics in Table 3). Collectively, these findings suggest that PME dysregulates BLA gene expression of opioid and dopamine receptors exclusively in adult females.

**Table 3:** Summary of statistics for opioid and dopamine receptor gene expression.

Target gene	Females	Males
*Gapdh*	*t* = 0.272, df = 15, *p* = 0.78	*t* = 1.109, df = 16, *p* = 0.28
*Oprm1*	*t* = 3.100, df = 15, *p* = 0.007**	*t* = 1.227, df = 16, *p* = 0.24
*Oprk1*	*t* = 0.067, df = 15, *p* = 0.94	*t* = 0.727, df = 16, *p* = 0.94
*Drd1*	*t* = 2.109, df = 15, *p* = 0.05*	*t* = 0.245, df = 16, *p* = 0.81
*Drd2*	*t* = 2.093, df = 15, *p* = 0.05*	*t* = 0.739, df = 16, *p* = 0.47
*Drd3*	*t* = 3.030, df = 15, *p* = 0.008**	*t* = 0.375, df = 16, *p* = 0.71
*Dat*	*t* = 1.029, df = 16, *p* = 0.31	*t* = 0.497, df = 15, *p* = 0.62

Bold values indicate statistically significant observations. *p < 0.05; **p < 0.01.

## Discussion

The primary goal of these studies was to determine whether PME might influence either baseline ethanol intake or stress-induced intake of ethanol, and the long-lasting neural consequences of PME. This is among one of the few studies investigating the long-term effects of PME on ethanol intake. The current study also assessed effects of PME on acute stress reactivity and cellular and molecular alterations within the BLA. In this study, we found that PME female offspring showed numerous alterations in all tested measures. Specifically, PME females consumed less ethanol than control females, in addition to showing increased immobility time in the FSS. Furthermore, BLA neurons from PME females had higher glutamatergic transmission, and there was dysregulation of gene expression for opioid and dopamine receptors within the BLA. Conversely, PME males only showed increased immobility time in the FSS test, but did not differ from control males in all other assessments. Collectively, these findings add to the growing literature demonstrating that PME leads to long-term alterations into adulthood and add further support to our previous observations that female offspring are particularly sensitive to PME’s effects.

Prenatal exposure to opioids may promote increased drug intake in offspring, thereby increasing the risk of substance use disorder (see reviews Abu and Roy, 2021; Grecco and Atwood, 2020). While various studies have tested effects of prenatal opioid exposure on future opioid or stimulant intake, whether prenatal opioid exposure alters future ethanol intake has only recently begun to be explored. One study by Grecco and Atwood found that pre-conception oxycodone exposure followed by PME in mice produced sex-specific effects on the rewarding properties of ethanol and ethanol intake in adolescent offspring (Grecco et al., 2022a). Specifically, PME males showed resistance to ethanol conditioned place preference, increased ethanol intake, and resistance to quinine-adulterated ethanol intake, while PME females exhibited enhanced sensitivity to the locomotor-stimulating effects of ethanol. Conversely, a different study reported that prenatal morphine exposure in rats did not affect ethanol intake (intermittent 2 bottle choice paradigm) in adolescent offspring (Parks et al., 2024), while unpublished work from Fleites and colleagues reported that prenatal morphine exposure in mice reduced ethanol intake (intermittent 2 bottle choice paradigm) in adult male offspring, but not female offspring (Fleites et al., 2022). Surprisingly, our findings indicate that PME reduced voluntary sweetened ethanol intake in a 30 min-limited access paradigm, but only in females. Furthermore, exposure to FSS ~10 h prior to the final drinking session did not affect ethanol intake, regardless of prenatal exposure. These findings were somewhat unexpected, since PME increased ethanol intake in adolescent offspring (Grecco et al., 2022a). However, it is possible that PME effects on ethanol intake may shift across development, as our findings are similar to those reported by Fleites and colleagues in prenatal morphine exposed offspring (Fleites et al., 2022). Although the mechanisms driving reduced ethanol intake in adult PME females are unknown, given that the ethanol solution was sweetened with sucrose and saccharin, it is possible that PME may produce anhedonia. While PME females showed increased immobility time during the FSS test compared to controls, which could suggest an increase in depressive-like behavior and support potential PME-induced anhedonia, it is worth noting that we used a single FSS session which has been suggested to reflect reactivity to stress more so than depressive-like behavior (Castagne et al., 2011). Thus, future studies should further assess this relationship and, in particular, how the sweetness of the ethanol solution plays a role in voluntary consumption. It is important to note that adult PME males also exhibited increased immobility time in the FSS test but had similar ethanol intake relative to control males; however, ethanol intake in all males was relatively low which may have prevented our ability to detect a further reduction in PME males. Another noteworthy difference between our study and that of Grecco and Atwood is the PME model. The PME model Grecco and Atwood used involved pre-conception exposure to oxycodone followed by a switch to methadone during the entire pregnancy with a higher dose of methadone (10 mg/kg) in mice, whereas our PME was limited to G3-20 with a max dose of 7 mg/kg in rats. Additionally, the assessment of ethanol intake differed between the two studies. Thus, there are several factors that may have contributed to differential effects on ethanol intake between the current study and the one by Grecco and Atwood. Regardless, given the limited number of studies testing the effects of prenatal opioid exposure on future ethanol consumption that, so far, have shown variable effects, there is a need for future studies examining this phenomenon.

One of the most interesting findings of the present study is that glutamate transmission was increased within the BLA of PME females relative to control females, with no differences evident in males. BLA pyramidal neuron activity has been correlated with anxiety-like behaviors (Wang et al., 2011), with infusion of glutamate antagonists into the BLA reducing anxiety-like behaviors (Lack et al., 2007). Thus, increased glutamate transmission in PME females could contribute to increased anxiety-like responses. In support of this, we previously found that adult PME females showed heightened freezing behavior across multiple extinction sessions in a contextual fear conditioning paradigm, whereas males did not exhibit these behaviors (Gamble and Diaz, 2023). It is possible that increased fear responding may be a result of a hyperactive BLA. As discussed above, PME females also had increased immobility time in the FSS test, which may indicate increased negative affective behaviors to which the BLA contributes. It is also interesting that only sEPSC frequency was increased in PME females, whereas sEPSC amplitude was not affected. Although there are many mechanisms that can contribute to the frequency of spontaneous neurotransmission, a change in frequency but not in amplitude may suggest a change in presynaptic function. There are numerous glutamatergic inputs into the BLA (see review Zhang et al., 2021), thus, it is possible that PME produces hyperexcitability of BLA inputs that may drive BLA activity. Future studies should further examine the impact of PME on other structures of the anxiety circuitry in addition to a more comprehensive investigation of the BLA microcircuitry. Importantly, the current study tested animals across a range of adulthood (P70-120) in order to align with the age of behavioral testing. Although it has been demonstrated that several neurophysiological mechanisms stabilize by P28 in the BLA (Ehrlich et al., 2013; Ehrlich et al., 2012), we acknowledge this may limit some interpretation of the data, and future studies should further examine if any age-related differences exist as a result of PME in the neurocircuitry assessed.

There are numerous neuromodulatory systems that regulate BLA function. Among these, the endogenous opioid and dopamine systems have been found to be a target of prenatal opioid exposure (Bhat et al., 2006; Darmani et al., 1992; Elam et al., 2022; Goetzl et al., 2019; Hou et al., 2004; McGinty and Ford, 1980; Robinson et al., 1997; Sarkaki et al., 2023; Wachman et al., 2018; Wang et al., 1986; Yazdanfar et al., 2021). Based on these studies, it is clear that there are numerous factors that influence the development of opioid and dopamine receptors, including the specific opioid exposure, timing of exposure, species, age of assessment, and brain region. Interestingly, our study found that PME females had reduced mRNA expression for the mu opioid receptor and dopamine D3 receptor, while also having increased mRNA levels for dopamine D1 and D2 receptors. We and others have previously shown that dopamine D1 and D3 receptors regulate GABA transmission in opposite ways in the BLA (Diaz et al., 2011; Diaz et al., 2014; Kroner et al., 2005), while dopamine D1 receptors modulate long-term potentiation within the BLA (Li et al., 2011). Dopamine inputs into the BLA also regulate anxiety-like behaviors in a variety of ways, depending on which dopamine receptor is activated or inhibited (Bananej et al., 2012; Brandao and Coimbra, 2019; Diaz et al., 2011; Zarrindast et al., 2011; Zhang et al., 2017). Mu opioid receptor activation within the BLA can both increase or decrease glutamate transmission depending on the mu receptor agonist used (i.e., morphine versus DAMGO) (Yang et al., 2014) and can regulate GABA transmission (Finnegan et al., 2006). Interestingly, BLA mu opioid receptors are involved in mediating motivation of cue-induced reward expectations (Lichtenberg and Wassum, 2017) and learning incentive values for food rewards (Wassum et al., 2011). Although our investigation into these neuromodulatory systems was only at the mRNA level, it is clear from our data that PME targets these two systems which may contribute to the observed changes in synaptic transmission and BLA-associated behavioral alterations. As with the behavioral and electrophysiological findings, it is quite interesting that the changes in gene expression were only observed in female offspring, while males did not show changes in any of the assessed genes. Nevertheless, it is important that future studies examine the impact of PME on opioid and dopamine systems within the BLA, both functionally and behaviorally, as this will elucidate the full extent of PME’s effects.

In conclusion, this is among the first studies to examine the long-term impact of PME on ethanol consumption. Additionally, this is also the first study that directly investigated PME effects on BLA physiology, which identified several potential mechanisms by which PME may produce maladaptive behaviors. Although PME reduced ethanol intake in adult female offspring, more studies are needed for assessment of PME effects on ethanol sensitivity and responsiveness later in life.

## Data Availability

The raw data supporting the conclusions of this article will be made available by the authors, without undue reservation.
